# Instantaneous Disturbance Index for Power Distribution Networks

**DOI:** 10.3390/s21041348

**Published:** 2021-02-14

**Authors:** María Dolores Borrás-Talavera, Juan Carlos Bravo, César Álvarez-Arroyo

**Affiliations:** Electrical Engineering Department, Escuela Politécnica Superior, Universidad de Sevilla, c/Virgen de África 9, 41011 Sevilla, Spain; carlos_bravo@us.es (J.C.B.); cesaralvarez@us.es (C.Á.-A.)

**Keywords:** power quality indices, signal processing, multi-resolution analysis, renewable energy applications

## Abstract

The stability of power systems is very sensitive to voltage or current variations caused by the discontinuous supply of renewable power feeders. Moreover, the impact of these anomalies varies depending on the sensitivity/resilience of customer and transmission system equipment to those deviations. From any of these points of view, an instantaneous characterization of power quality (PQ) aspects becomes an important task. For this purpose, a wavelet-based power quality indices (PQIs) are introduced in this paper. An instantaneous disturbance index (ITD(t)) and a Global Disturbance Ratio index (GDR) are defined to integrally reflect the PQ level in Power Distribution Networks (PDN) under steady-state and/or transient conditions. With only these two indices it is possible to quantify the effects of non-stationary disturbances with high resolution and precision. These PQIs offer an advantage over other similar because of the suitable choice of mother wavelet function that permits to minimize leakage errors between wavelet levels. The wavelet-based algorithms which give rise to these PQIs can be implemented in smart sensors and used for monitoring purposes in PDN. The applicability of the proposed indices is validated by using a real-time experimental platform. In this emulated power system, signals are generated and real-time data are analyzed by a specifically designed software. The effectiveness of this method of detection and identification of disturbances has been proven by comparing the proposed PQIs with classical indices. The results confirm that the proposed method efficiently extracts the characteristics of each component from the multi-event test signals and thus clearly indicates the combined effect of these events through an accurate estimation of the PQIs.

## 1. Introduction

The extensive use of power-switching devices for source conditioning, renewable energy supply and motion control in modern industrial applications has detrimental side-effects on power quality, such as increase potential for unacceptable harmonic levels, poor power factor, or unbalanced currents and voltages in power distribution networks. In addition, transients caused by faults and switching events in power systems significantly affect the power-transfer quality of a supply [1,2]. All these undesirable effects cause huge economic losses [3] and require an effective power quality analysis in the grid and affected facilities. Smart sensors proposals are rapidly increasing in response to these new requirements [4,5,6,7,8,9]. Although the first applications were almost exclusively limited to billing, they now include new features related to power quality detection and have a common denominator: they require high processing speed to handle large computer data in a shorter time. In this context it is crucial to provide a small number of parameters and indicators that can effectively characterize all these events and derive from a very fast and efficient analysis tool. This task is the main objective of this work.

The power quality index (PQI) is the summary of waveform distortions in voltage and current from the perfect sinusoids. The PQIs are used to characterize the degree of quality degradation in a quantitative manner. They represent the impacts of non-ideal waveforms on electrical power systems in a compact but expressive manner. Existing indices such as Total Harmonic Distortion (THD), Power factor, Flicker factor, etc. reflect the degree of power disturbance in each of these categories individually, but fail to assess most of the phenomena mentioned together in an exhaustive and concise manner by a single value [10].

Several methods have been applied to research the real-time behavior of controllers, test and protection equipment [11] and fault diagnosis devices [12,13]. All of these applications, together with those mentioned above, have a positive and economic impact on the industry. A measuring device integrated into the system offers the additional characteristic of quantifying the various characteristics that affect power quality and thus identifies any specific aspect that needs attention.

Power quality measurement and analysis has typically been divided between steady-state concerns, such as harmonic distortion, and transient concerns, such as those resulting from faults or switching transients. Fourier analysis has been applied to the first class of problems while Joint Time-Frequency Analysis (JTFA), including wavelets, has traditionally been used for the second class [14].

When using the discrete Fourier transform (DFT) in nonstationary situations, the estimation of a time-varying signal during a specific time interval results in a serious deterioration of measurement accuracy. The effect is equivalent to the frequency deviation of the power system from its nominal value when measuring electrical signals [15,16]. To avoid this problem, the Fourier Transform applied in time segments as the Short-Time Fourier Transform (STFT) determines the frequency contents of a signal in each time window. Nevertheless, the size of the window affects the capacity of multi-resolution capability and the cost of the calculations is high. These deficiencies can be solved by using the Discrete Wavelet Transform (DWT) that also avoids the problems of interference of time and frequency distributions [17]. Besides, the DWT method offers a better time and frequency resolutions for high and low-frequency components, respectively. This method is best for locating disturbances in non-stationary conditions. However, the limitation of the DWT uncertain can be a shortcoming that can be minimized by an appropriate choice of the analyzing wavelet basic function. In this article, the Daubechies40 wavelet offers the best results for the analyzed electrical signals present in power distribution networks.

Traditionally, DWT [18,19,20,21,22,23,24], the generalized S-transform (GST) [25,26,27,28,29,30], the Time-Frequency distributions, and the Short-DFT (SDFT) [31] have been used for the analysis of the transient and time-varying nature of disturbance signals in electric power systems. In particular, power quality indices [32,33,34,35,36,37,38] have been defined using these transforms. Thus, the estimation of standard time-varying PQIs is done based on a proper JTFA [32], adaptive window-based fast generalized S-transform [33], empirical wavelet transform (EWT)-based time-frequency technique [34], cluster analysis of long-term power quality (PQ) data [35], wavelet packet transform [36], load composition rate and Euclidean norm of total harmonic distortions [37], and global harmonic parameters for phasor measurement units [38]. Most of these works use many PQIs in their proposals, for example, the global PQIs introduced in [35] contain up to seven PQI factors. As will be shown later, only two PQIs are used in the present work. Specific treatment of transient events is done in [39], whereas in [37] only stationary distortion is considered. Thus, it is necessary to conceive a measure of power quality in order to capture simultaneously both the “transient” characteristics of disturbance signals in electrical power systems [39] and the stationary ones. Moreover, it must be done with a minimum computational cost and using a single descriptive indicator. Wavelet-based techniques are good candidates for this purpose. However, a method based on empirical wavelet transform will work properly for off-line data processing but not for real-time analysis due to its computational cost [34,40].

Due to their powerful characteristics, in this work, DWT and multiresolution analysis (MRA) are chosen for the joint analysis of the stationary and transient parts of electrical signals. These parts are extracted from a monitoring window to ensure the correct use of the DWT. In this way, the fundamental component of the electrical signal is extracted and the window containing the transient disturbance is processed. Both aspects are subsequently used to construct new DWT based power-quality indices that replace existing counterparts. On the other hand, the main drawback of this method is based on its limitation in the analysis of highly noisy signals. In these noisy conditions, the method has been proven to work properly until the minimum value of 34 dB for the signal-to-noise ratio (SNR) [19], which is more than enough for most of practical PQ events detections in low-voltage AC power distribution networks.

Reference [41] presents an interesting overview of power quality in low-voltage DC distribution networks. This study highlights the most relevant disturbances in DC networks and how they can be accurately characterized by means of power quality indices, some of which have been defined based on DFT. Due to the close relationship between the indices defined in AC power system networks and DC networks, a characterization of PQ in DC networks using the current DWT-based method seems to be interesting and deserves future work. In this possible context, the implementation of the algorithms derived from the present work in smart sensors may provide new functionalities for PQ improvement. However, they could increase the power consumption requirements of such sensors. In this way, reference [42] presents a useful study of the power requirement of functional sensors in a traditional PV system. It is based on Neural Network maximum power point tracking with cloud method and estimates the reduction percentage of power consumption in functional sensors.

In this paper we propose two wavelet-based power quality indices, the Instantaneous Disturbance Index (ITD) and the Global Disturbance Ratio (GDR), which comprehensively assess the power transfer quality of a given supply in steady-state and/or transient situations. The proposed ITD instantly shows the evolution of the PQ. It has the advantage of evaluating the PQ in real-time situations under any conditions and extracting the characteristics of the disturbance for any load in the power distribution networks. In addition, GDR has the advantage of assessing PQ by means of a single value and allows distinguishing between different events, so the GDR can be used as an input in a disturbance classifier. Therefore, the new PQ indices can identify properly practical waveform distortions in power networks. They can also be used to evaluate both the effectiveness and dynamic responses of PQ mitigation equipment in practical applications. In this work, a real-time platform is being developed to experimentally validate the feasibility of the proposed PQI measurement and analysis method.

The rest of this document is organized as follows: In Section 2, a simplified outline of wavelet filter selection criteria is presented. Section 3 briefly describes the DWT-based instantaneous indices used to evaluate PQ. Section 4 shows a detailed summary of the proposed measurement process is shown. In Section 5, results are discussed. The last section draws conclusions from the results.

## 2. Fundamentals of the Proposed Indices

### 2.1. Components Signal Estimation

The proposed estimation technique uses DWT and MRA for extracting the fundamental component of the input signal *s*(*t*), which can be described by wavelet coefficients [14,24]

(1) $$s(t)=\sum_{k=1}^{2^{J}}a_{J,k}\;\phi_{J,k}(t)+\sum_{j=1}^{J}\sum_{k=1}^{2^{J}}d_{j,k}\;\psi_{j,k}(t)$$

where *j* and *k* are the wavelet frequency scale and wavelet time scale, respectively, *J* is the highest *j* scale, i.e., the lowest frequency band; *a_J,k_* and *d_J,k_* are the wavelet coefficients; and *ψ*(*t*) and *φ*(*t*) are the mother and scale wavelet functions, respectively. Equations (1)–(3) present the basic concepts of MRA. The aim of MRA is to develop representations of a complex signal *f*(*t*) in terms of scaling and wavelet functions.

The digitized version of input signal *s*(*t*) is a sequence of *n* samples, *s*(*n*), which can be processed in the same way as the DFT. In addition, the number of DWT levels (decomposition) is limited by the number of the original signal samples, which in turn must be a power of two.

Therefore, signal *s*(*n*) can be presented in terms of its frequency components, i.e., coefficient *a_J,k_*, *k* = 1, …, 2^*J*^, is the smoothed version of signal *s*(*n*), and coefficients *d_j,k_*, *k* = 1, …, 2^*J*^, *j* = 1, …, *J* are detailed versions of *s*(*n*). They contain the lower and higher-frequency components, respectively [14].

(2) $$s(n)=a_{J}(n)+\sum_{j=1}^{J}d_{j}(n)$$

With

(3) $$\begin{array}{l} a_{j}(k)=\sum_{n}g(n-2k)a_{j-1}(k)\\ d_{j}(k)=\sum_{n}h(n-2k)a_{j-1}(k) \end{array}$$

where *a_j_* and *d_j_* are the approximation and detail coefficients at level *j*, *g*(*k*) and *h*(*k*) are the high pass and low pass filters corresponding to the scaling and wavelet filters. The coefficients have half of the original input data due to the downsampling process. 

For extracting the fundamental component of a signal by using MRA, the sampling rate and, so, the number of MRA steps must be specified. If it is assumed that *H* is the frequency band order with central frequency, *ω*/(*2π*), equal to fundamental frequency and *fs* is the sampling frequency, the number of MRA steps, *J*, satisfies the following expression:

(4) $$\frac{fs}{2^{J}}=2\times H$$

In this work has been assumed that the most important transients occurring in actual situations of Power Distribution Networks (PDN) are captured into a frequency band of 6400 Hz, for *fs* = 1/*T_S_* = 12.8 kHz. It assures accurate results with *J* = 6.

### 2.2. Decomposition Structures of MRA Method

MRA method uses a decomposition structure based on Quadrature Mirror Filter (QMF). QMF consists of two complementary filters, one low pass and the other high pass, which disjoin the frequency range into two equal parts. They decompose the input signal into two frequency intervals, the low pass filter output is downsampled and is used as new input of another identical filter pair corresponding to the next decomposition level. This operation is repeated recursively, decomposing the signal into approximation (*a*) and detail (*d*) coefficients for various scales.

The coefficients of the scale *g* and wavelet *h* filters, and the efficiency of wavelet analysis, are related to the selected mother wavelet. The right selection of the mother wavelet is the main task to obtain the desired results in signal analysis with WMRA. In this work, the selection of the analyzer function is based on the efficiency of the scale and wavelet filters used for this purpose.

**Figure 1:**
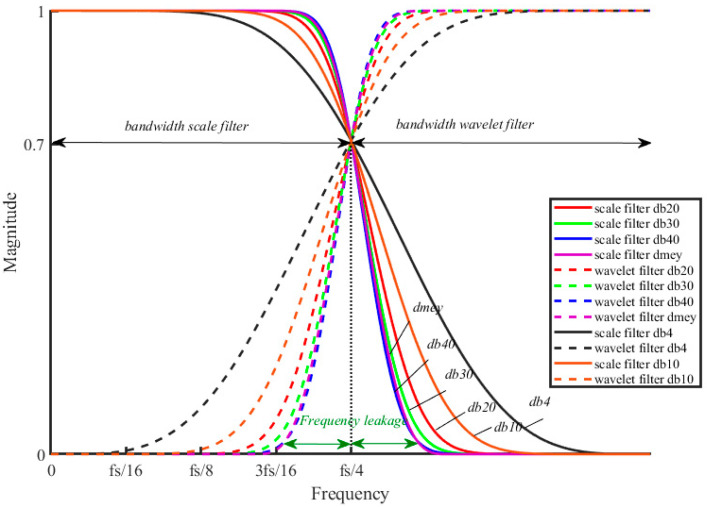
Comparative frequency response of mother wavelets.

The QMF filters frequency response of the first level DWT decomposition is shown in Figure 1. It depicts a comparison of the scale and wavelet filters between the db4, db10, db20, db40, and dmey mother wavelets used on the MRA method. The filters have overlapping frequency bands and energy leakage occurs between the two adjacent bands affecting the obtained coefficients. It is less pronounced with db40 and dmey mother wavelet.

### 2.3. Wavelet Filters Selection

Several mother wavelets have been evaluated in order to select the most suitable for the specific application of event detection and classification methods. In this paper, the selection of an explicit wavelet is based on the following principles:

- Minimum frequency leakage of QMF in the first levels of decomposition.
- Number of filter coefficients.
- Similarity between classical THD (Total Harmonic Distortion Ratio) with the TWD (Total Wavelet Disturbance Ratio) defined with wavelets.

The main selection criterion is the frequency selectivity. It is based on the magnitude transition zone slope of the frequency response of the wavelet filters. The selected mother wavelet will have better characteristics if the slope of both filters has the highest value and the information dispersion related to the frequency content of the signal will be less. In this way, a greater concentration of energy is obtained in single frequency bands.

A comparison between Fourier and Wavelet analysis is made to select the most appropriated. The STFT has good performance with signals disturbed by harmonics. According to the Parseval theorem, the energy of a signal can be decomposed in terms of the energy of *a* and *d* coefficients, the selected mother wavelet will be that allocate the signal energy correctly. 

**Figure 2:**
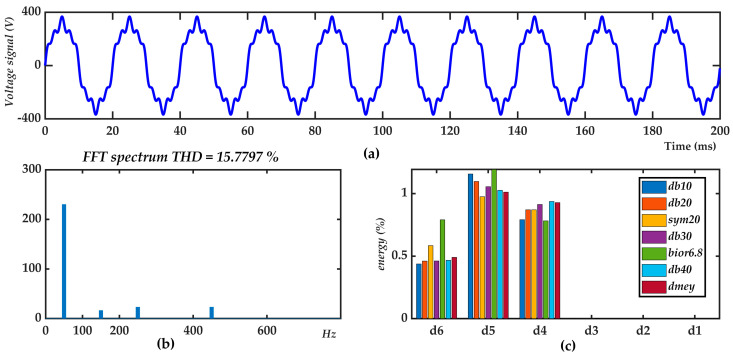
Voltage signal with harmonics: (**a**) Instantaneous representation, (**b**) FFT spectrum, (**c**) distribution of the energy percentage of the signal according to the coefficients of the mother wavelets.

Figure 2a shows a signal that contains three harmonics, the 3rd (7% Urms), the 5th (10% Urms), and the 9th (10% Urms). This signal is chosen to support the selection of the best mother wavelet available. Figure 2b shows the FFT spectrum where the fundamental harmonic has 97.570% of the total signal energy, the 3rd has 0.478% and both the 5th and 9th have 0.976%. Figure 2c also shows as a bar graph the energy percentage of this disturbed signal obtained with various mother wavelets. Table 1 shows the percentage of signal energy achieved with the mother wavelets depicted in the legend of Figure 2c. 

Table 2 shows the THD and TWD results calculated, the results are similar excluding bior6.8 and db10 ones.

According to Table 1, the mother wavelets with the best frequency response correctly distribute the energy of the signal according to the FFT spectrum (Figure 2). Therefore, the db40 and dmey wavelets are the best mother wavelets. Since the db40 wavelet has fewer coefficients than the dmey wavelet, the db40 is the mother wavelet with the best performance.

## 3. Quantitative Formulations of Steady-State and Transient Power Quality Aspects

This formulation is based on the IEC 61000-4-7 standard [43], EN-50160 standard [44] and the IEEE Std 1159™-2009 [45] guidelines, in order to fulfil the essential requirements and to define the characteristics of the supplied voltage and the most common disturbances. 

### 3.1. DWT-Based Disturbance Ratio

The Total Harmonic Distortion ratio (*THD*) for single-phase networks (or polyphase balanced networks) has been defined traditionally as [43,46]

(5) $$THD=\frac{\sqrt{\sum_{h=2}^{H}S_{k}^{2}}}{S_{1}}\cdot 100\;H=\frac{f_{m}}{2\cdot f_{0}}$$

(6) $$THD=\frac{\sqrt{S^{2}-S_{1}^{2}}}{S_{1}}\cdot 100$$

where *S* denotes RMS value of signal *s*(*n*), *S*_1_ denotes fundamental component of *s*(*n*), and *S_k_* the *k* component of *s*(*n*). Using the MRA tool, *S* is given by

(7) $$S=\sqrt{Sa_{J}^{2}+\sum_{j\leq J}Sd_{j}^{2}}$$

Here *Sa_J_*, is the RMS value of the N samples signal *a_J_*(*n*) in the lowest frequency band *J*, where the fundamental component *S*_1_ is included. {*Sd_j_*} is the set of RMS values of *d_j_*(*n*) signal in the higher frequency band, or wavelet-level lower than or equal to the scaling level *J*. Then, the Total Wavelet Disturbance ratio *TWD* [47] is defined as

(8) $$TWD=\frac{\sqrt{\sum_{j\leq J}Sd_{j}^{2}}}{Sa_{J}}\cdot 100$$

### 3.2. DWT-Based Instantaneous Disturbance Ratio

The Instantaneous Transient Disturbance ratio (*ITD*(*n*)), the transient version of *TWD*, is defined (9) in terms of the time-scale distribution of the MRA components:

(9) $$ITD(n)=\frac{\sqrt{\sum_{j\leq J}d_{j}^{2}(n)}}{A_{J}}\cdot 100$$

where *A_J_* is the fundamental energy component defined as:

(10) $$A_{J}^{2}=\frac{1}{N}\sum_{n=1}^{N}a_{J}^{2}(n)$$

It can be seen that *Sa_J_* is identical to *A_J_*.

The definition of the *ITD*(*n*) can be interpreted as a “time-varying” power quality evaluation determined by the time-frequency localized energy ratio of the disturbance events to the fundamental frequency energy.

As an instantaneous quantity, the proposed *ITD*(*n*) index can reveal the time-varying characteristics of the transient disturbance for assessment purposes. 

The time-varying signature can be quantified as a single number, as in the case of steady-state disturbances. Therefore, a “transient-interval average” of the *ITD*(*n*), <*ITD*>, can be defined over a sample interval *N* as follows:

(11) $$\langle ITD\rangle =\frac{1}{N}\sum_{n=1}^{N}ITD(n)$$

Note that <*ITD*> is almost identical to *THD* when only steady-state disturbances are presented in the signal. 

The non-stationary events duration is a very relevant parameter to be considered. It can be measured with high precision by the wavelet procedure used in this work. Then a Global Disturbance Ratio, *GDR*, can be defined,

(12) $$GDR=\left(1+\frac{T_{0}}{T}\right)\langle ITD\rangle$$

where *T*_0_ is the duration of the transient disturbance and *T* is the time interval window used. The selection of the time interval (*T*_0_) can be determined by the time index of the first maximum peak value of the *ITD*(*n*), *t*_0_, and the time index of the last maximum peak value of the *ITD*(*n*), *t*_0_ *+ T*_0_. Only if steady-state disturbances are present in the signal, *GDR* = <*ITD*>, otherwise *GDR* is greater than <*ITD*>.

Consequently, the proposed index *GDR* is the transient-interval average, <*ITD*>, plus its magnitude weighted by a term related to the event duration. 

A loaded power network with sinusoidal voltages yields an ideal null *GDR*. Conversely, a high value of *GDR* would indicate a high level of steady-state and/or transient disturbances, with the contribution of each event aspect well defined and measured. Note that the proposed index *GDR* presents the advantage over the *THD* of distinguishing transients and stationary events. The duration of the disturbance plays a significant role in the *GDR* index.

## 4. Measurement Process

### 4.1. Developed Platform

**Figure 3:**
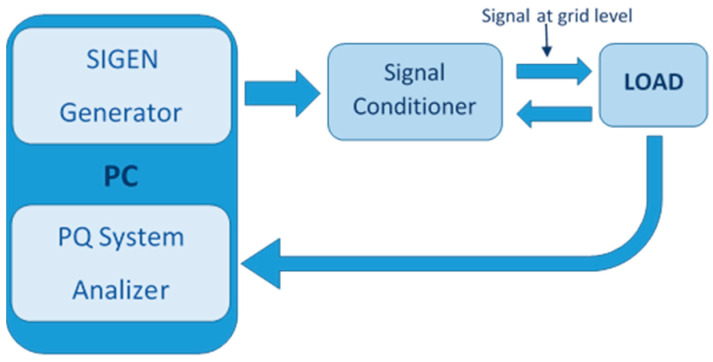
Developed system scheme: Voltage generator with preset disturbances, power amplifier, and signal conditioner, load and Power Quality Analyzer.

In this work, a developed platform is used to test the effectiveness of the proposed indices under common real-time working conditions (Figure 3). This procedure permits the emulation of actual power systems. In this context, the proposed instantaneous indices are suitable.

The developed system consists of a signal generator that allows the design of any kind of disturbance. This signal is amplified, conditioned and applied to a real load. Finally, the voltage and current are measured on the load side and processed in a power quality analyzer.

**Figure 4:**
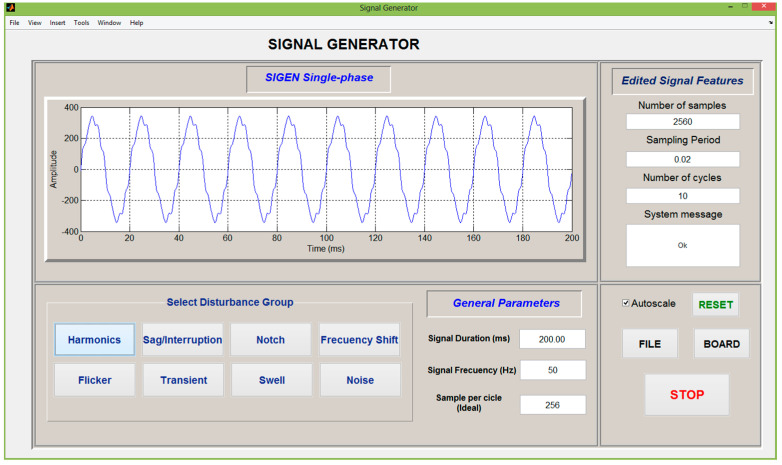
Graphical user interface of the *Sigen* system.

Matlab^®^ software has been used to program a virtual signal generator, called *Sigen*, which allows a complete configuration of the signals required for testing. *Sigen* mainly generates a pattern based on the parameters defined by the user; thus, steady-state and/or transient-state disturbances can be modelled. The Graphical user interface of single-phase *Sigen* is shown in Figure 4.

The processes of *Sigen* are performed to complete the effective generation of electric signals, as follows.

- First, electrical inputs signals are defined and the user sets their parameters.
- Second, signals according to these specifications are built.
- Finally, the designed signals are sent to a file or to the data acquisition board (DAQ).

*Sigen* is designed to program disturbances as described in IEEE standard 1159-09 for monitoring electric power quality [45].

The design is based on generating single or three-phase voltages and line currents. Generated data sets are obtained from a host PC in data files with the American Standard Code for Information Interchange format compatible with the most popular data analysis tools.

The host PC is equipped with the NI USB-6259; it is a 16-Bit High-Speed M Series Multifunction DAQ for USB. It acquires eight differential inputs. Analog inputs are converted with 16 bits of resolution sampled at 1.25 MS/s. Voltage and current sensors are built with Hall Effect voltage and current transducers, type LV25-P and LA25-NP, respectively. Low-level voltage signals proportional to the phase-neutral voltages are available. 

At the final stage, an amplifier section increases the voltage signals up to the grid voltage level. A decisive request remains on the amplifier section since it must ensure accurate and constant gain and phase shift overall bandwidth required. A Pacific Power Source Model 320 is used as a power amplifier to fulfill all the proposed requirements (output voltage up to ±600 peak volts; maximum output power: 1.2 kVA; bandwidth (30–5 kHz) at full power; THD < 0.2%).

Disturbed voltage signals at the grid level are generated for studying Power Quality Events in several types of loads.

The generated voltage signals are used to simulate a power system with actual voltage sources and arbitrary loads. It can process polyphase sinusoidal voltages added with simple or multiple disturbances.

**Figure 5:**
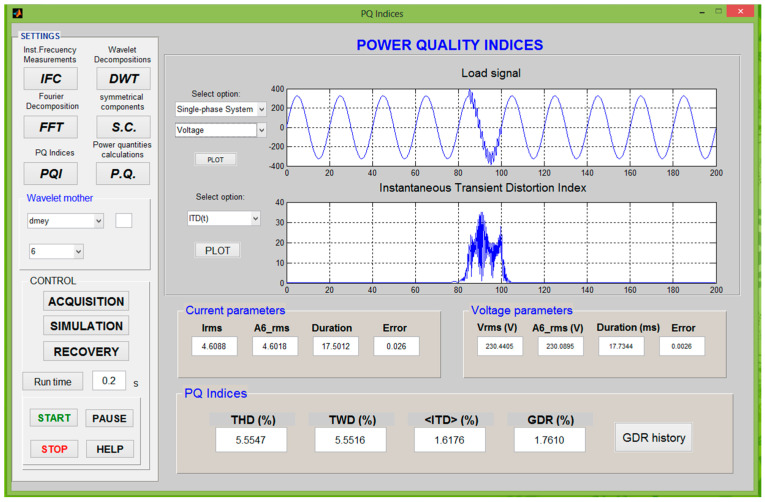
Graphical user interface of the power quality (PQ) System Analyzer.

The power-quality analyzer is a virtual device that processes the signal data file from an A/D converted connected to the signal conditioner (Figure 5). A control program developed in MATLAB^®^ diagnoses quality aspects of the input signals, such as frequency stability, distortion level, symmetry of three-phase signals (balance between phases R, S and T), and others that can be inferred from the graphical user interface of Figure 4.

In order to carry out this diagnosis, the system can measure and present/display the graphs (with its time evolution):

- Instantaneous network frequency, following its changes at intervals of measurement of one cycle, considering deformed signals and with adding noise.
- Harmonics, represented in phasor form using two bar charts, one for magnitudes and another for phases.
- Instantaneous PQI and coefficients of power quality indices (percent), and a presentation of the data corresponding to the signals.
- DWT coefficients with a representation of the wavelet level selected by the user.
- Three-phase signals of voltage and current, or in its place, their respective fundamental symmetrical components, i.e., the fundamental components of positive, negative and zero sequences.

Furthermore, the PQ System analyzer computes power quantities in Wavelet and Fourier domain specified by IEEE standard 1459–2010 [46].

The developed platform and the proposed indices can be further used in real-time for both monitoring and detecting faults in power networks [20] and electrical machinery [48]. By means of a signal-based fault diagnosis method, the proposed novel indices can be applied to loads such as induction motors, power converters, and mechanical components.

### 4.2. PQI Measurements

Different kinds of power quality disturbances are applied through the power voltage amplifier to linear and nonlinear loads. In these loads, voltages and currents are taken by the PQ System Analyzer.

For the voltage quality assessment, instantaneous frequency measurement is performed which enables synchronization between the signal period and the sampling sequence. 

For the considered voltage and current windows specified by IEC standard 61000-4-30 [49], time-frequency-based quality aspects are calculated by the DWT. For the case of 12.8-kHz sampling rate in the *Sigen* system, Table 3 summarizes the frequency band information for different wavelet analysis levels. The db40 mother wavelet is applied in MRA of the voltage and current signals.

According to Equations (5), (6), (8), (9), (11) and (12) the PQIs are computed. The PQ indices have been tested on a variety of stationary, non-stationary single-phase signals as well as balanced or unbalanced three-phase signals.

To measure properly disturbances with a higher duration than the considered window, the PQ system analyzer provides a *GDR* history tool to save stored data of the *GDR* index.

The fundamental component of the grid voltage presented in all the used test signals is distorted by harmonics and/or non-stationary events, in accordance with both, the IEEE standard 1159-2009 [45] and the European standard EN-50160 [44].

## 5. Illustrative Results

To show the effectiveness of *GDR* and <*ITD*>, *TWD*, and *THD* indices are also calculated and the result obtained has been compared in two examples sets. In all of them, the grid voltage waveform contains a fundamental component of 230 Vrms, 50 Hz of nominal frequency, and stationary and/or transient disturbances. In the first set, a group of signals with single disturbances and almost identical *THD* index are considered. In the second set, complex signals with more significant and combined disturbances are studied. 

### 5.1. Voltages Waveform with Similar THD

**Figure 6:**
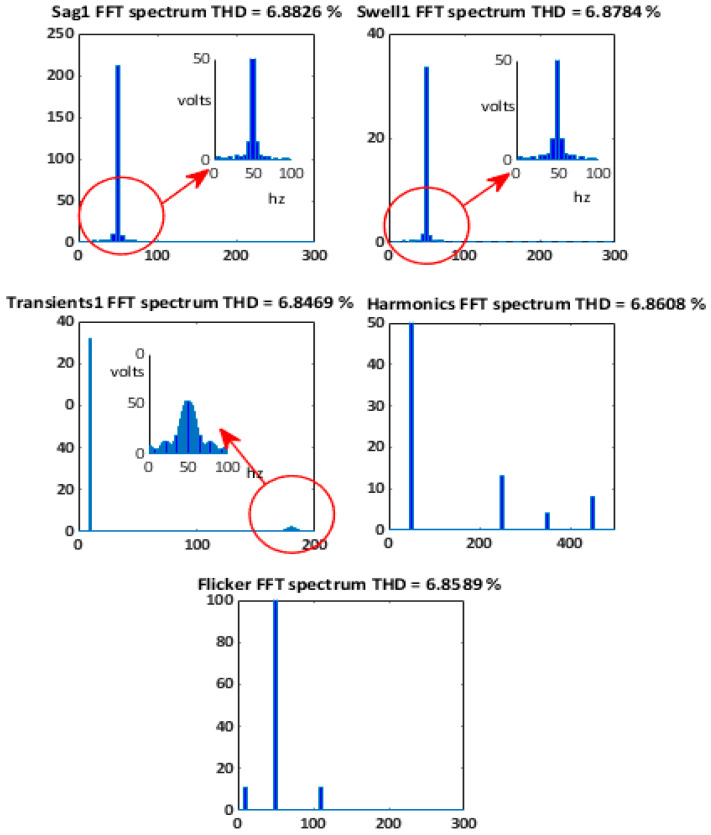
FFT spectra for several signals used on case A.

**Figure 7:**
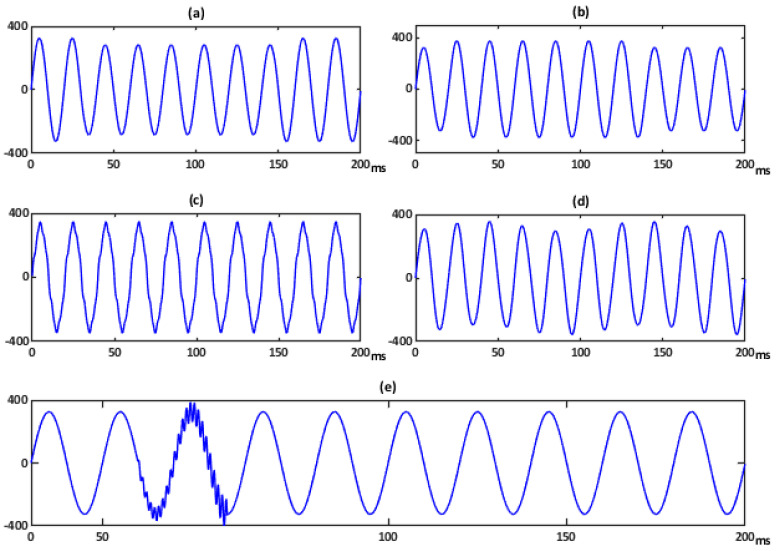
Single-phase voltage disturbances with similar THD. (**a**) Voltage sag1. (**b**) Voltage swell1. (**c**) Harmonic disturbances. (**d**) Flicker disturbance. (**e**) Transient1 disturbance.

Several disturbed voltage waveforms extracted from the developed platform are analyzed. In particular, the considered signals are three voltage sags and two swells with different duration and amplitude, three oscillatory transients with distinct frequency and duration, a steady-state distorted voltage with three harmonics (5th, 7th, and 9th with relative amplitudes depicted in the FFT spectrum of Figure 6) and a flicker disturbance. Some of them are depicted in Figure 7 and they all only have in common a similar THD value.

**Figure 8:**
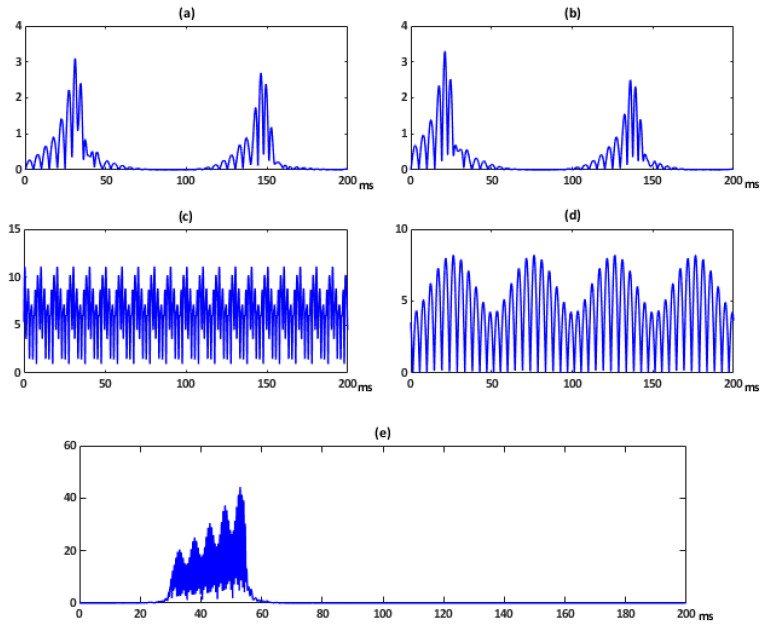
Instantaneous Transient Disturbance ratio index *ITD*(*t*) of voltage disturbances corresponding to Figure 5.

These voltage signals are applied to linear and nonlinear loads, and the resulting load currents are analyzed in our PQ System Analyzer too. The corresponding voltage FFT spectra are shown in Figure 7. The *ITD*(*t*) of the signals shown in Figure 6 are respectively depicted in Figure 8. The first *ITD*(*t*) at the left top of Figure 8 shows two peak values: 3.1% at 31.1 ms and 2.7% at 146.2 ms. The second *ITD*(*t*) at the right top shows two peak values: 3.3% at 21.2 ms and 2.5% at 136 ms. The peak values in both signals are associated with the depth/crest of the corresponding sag/swell. The *ITD*(*t*) attaching to oscillatory transient of Figure 8 shows a peak value of 44.27% at 53 ms. In this context, the peak values indicate that the oscillatory transient is a more severe event than the harmonic distortion. However, it presents the shortest time duration and the highest frequency content of the five disturbances (FFT spectra of Figure 7). The *ITD*(*t*) for steady-state conditions do not offer any new relevant information over the *THD*, nevertheless <*ITD*> is almost identical to *THD* (Table 4). In this case, the error between both quantities is less than 0.5%.

Table 4 provides the quantities obtained for all the examples discussed in terms of their respective duration (*T*_0_), Vrms value and time-frequency based transient power quality indices. It can be seen that *THD* is almost the same for all disturbed signals even if they correspond to different classes of disturbances. *TWD* is similar to *THD* in those signals in which the frequency bands are far from the fundamental energy component. On the contrary, in those with the frequency components near to the fundamental, the values are very different and the duration of the disturbance does not make any substantial difference. Instead, *GDR* and <*ITD*> assessments are more consistent with the energy content of these disturbance signals.

The *ITD*(*t*) (Figure 8) shows the instantaneous character of the disturbance noting the relevance of any suddenly introduced change. Instead, its averaged energy over the observation window is considered by the *GDR*. Furthermore, the *GDR* permits to distinguish between different disturbances and can be used for classification purposes. In particular, the *GDR* differentiates the proposed disturbances, with almost the same *THD,* by giving importance to both, the amplitude and the duration of the disturbance. This index offers the advantage to indicate clearly the most relevant of them, as can be seen in Table 4 for any kind of the disturbances numbered as “1”. So transient events are perfectly characterized by this procedure. Nevertheless, the studied sag and swell signals have the same frequency contents and are undistinguished by all the indices, so the RMS value has to be considered as an assisting index.

### 5.2. Disturbances Combination in Voltage Waveforms 

In practical situations, a PQ event usually consists of a combination of singular disturbances, most of them treated in case A. Therefore, a set of complex disturbed signals with more severe and combined effect are studied. 

**Figure 9:**
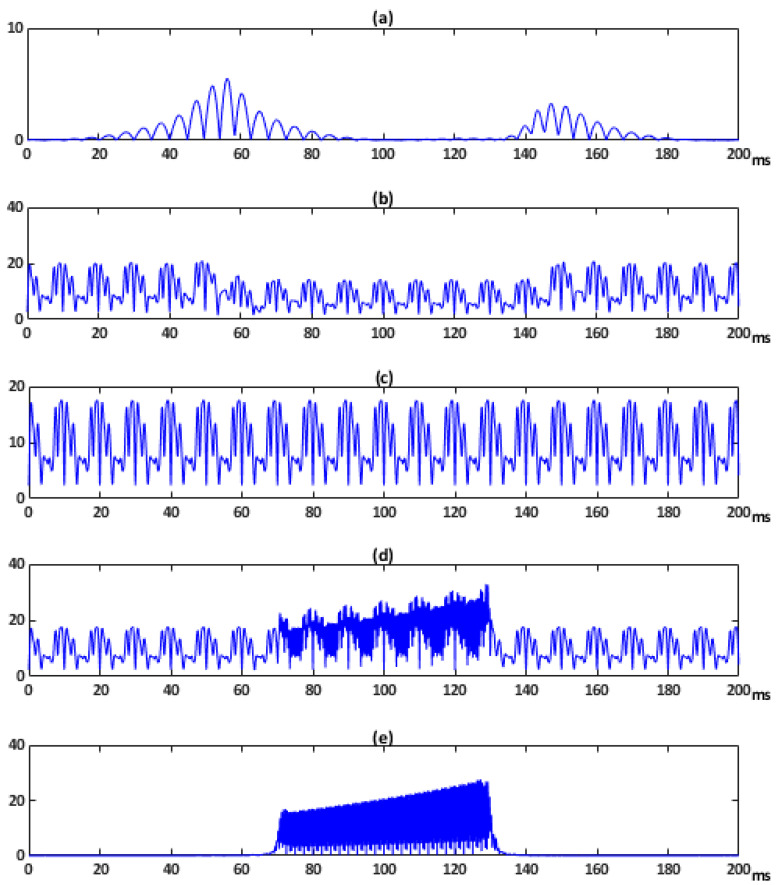
Instantaneous Transient Disturbance ratio index *ITD*(*t*) of voltage disturbances combination.

Figure 9a depicts the *ITD*(*t*) corresponding to a pronounced sag. It shows two peak value: 5.5% at 56.25 ms and 3.3% at 147.25 ms. Figure 9b shows this index for a combination of the mentioned sag and a steady-state distorted voltage with five harmonics (3rd, 5th, 7th, 9th, and 11th and Vrms equal to 10 V, 17 V, 7 V 13 V, and 3 V, respectively). The *ITD*(*t*) resulting from a steady-state distorted voltage only with the five harmonics is provided in Figure 9c. In addition, Figure 9d shows this index for a combination of the same steady-state voltage and an important transient oscillation. The *ITD*(*t*) of a voltage disturbed only with this single transient is shown in Figure 9e. It has a peak value of 30% at 127 ms.

In this case, the waveform offers more relevant changes than the previous set of examples because more substantial disturbances are present. When the signal is disturbed with two events, both are evinced in *ITD*(*t*) graph.

The PQ indices obtained are presented in Table 5. By comparing the values obtained, the *GDR* index remarks the effect of the disturbances combination. As it can be observed from Table 5, the proposed index is the only one capable to indicate clearly the accumulative effect of the combined events in complex signals. Although the *GDR* index has not the additivity property, it performs the indication by significantly increasing its relative value when distinct disturbances are present.

## 6. Conclusions

The motivation for this work stemmed from the growing need for a more effective analysis of power quality in electrical systems and equipment. In this way, the main contribution of this research is the introduction of a novel wavelet-based single indicator, designated global disturbance ratio (*GDR*) that has been tested on real signals to address an integral assessment of the electrical network PQ. The objective of these test is to guarantee the applicability of such index to smart network sensors in particular, and to PQ monitoring in general. To this end, a PQ System Analyzer based on wavelet techniques is developed, turning out to be an effective device to verify the behavior of the proposed indices. 

The *GDR* is based on an instantaneous index *ITD*(*t*), which is also introduced, and considers two quality aspects of the electrical signal: steady-state power quality relative to harmonic level, and the non-stationary index relative to oscillatory transients or sudden amplitude changes in the signal. 

In the initial theoretical stage of signal analysis it is remarkable the mother wavelet selection procedure used to guarantee the most accurate frequency decomposition of the voltage signal under steady-state and non-stationary events.

The applicability of the *GDR* and *ITD*(*t*) is illustrated for typical source-load configurations. The proposed PQIs have been tested on single-phase stationary and non-stationary complex signals, as well as three-phase balanced and unbalanced signals.

Finally, the *ITD*(*t*) and *GDR* indices are a powerful tool for detecting and monitoring non-stationary signal components and they can extract relevant characteristics. In particular, the *GDR* index may be applied as the input of a classifier. In this sense, the authors are working on the physical implementation of an intelligent sensor based on a DSP, which provides the proposed PQIs. This guideline deserves future research.

## Figures and Tables

**Table 1:** Energies (%) of voltage signal decomposition shown in Figure 2.

f(Hz)	a6 0–100	d6 100–200	d5 200–400	d4 400–800	d3 800–1600	d2 100–200	d1 3200–6400
db10	97.6142	0.4378	1.1561	0.7912	0.0007	0.0000	0.0000
db20	97.5719	0.4610	1.0966	0.8706	0.0000	0.0000	0.0000
sym20	97.5705	0.5844	0.9745	0.8706	0.0000	0.0000	0.0000
db30	97.5704	0.4616	1.0550	0.9130	0.0000	0.0000	0.0000
bior6.8	97.2315	0.7903	1.1931	0.7817	0.0035	0.0000	0.0000
db40	97.5699	0.4673	1.0257	0.9371	0.0000	0.0000	0.0000
dmey	97.5705	0.4905	1.0116	0.9274	0.0000	0.0000	0.0000

**Table 2:** Total Harmonic Distortion (THD) and Total Wavelet Disturbance Ratio (TWD) of voltage signal decomposition shown in Figure 2.

THD	TWD db10	TWD db20	TWD sym20	TWD db30	TWD bior6.8	TWD db40	TWD dmey
15.7797	15.6335	15.7751	15.7798	15.7801	16.8741	15.7818	15.7796

**Table 3:** Frequency bands and harmonics of six levels of the Discrete Wavelet Transform (DWT).

Level	Freq. Band (Hz)	Odd Band Harmonics
7 (d1)	3200–6400	63rd–127rd (odd num.)
6 (d2)	1600–3200	33rd–63rd (odd num.)
5 (d3)	800–1600	17th–31st (odd num.)
4 (d4)	400–800	9th, 11th, 13th, 15th
3 (d5)	200–400	5th, 7th
2 (d6)	100–200	3rd
1 (a6)	DC-100	1st

**Table 4:** Summary of Power Quality Indices for the case A.

	Sag1	Sag2	Sag3	Harmonics	Transient1	Transient2	Transient3	Flicker	Swell1	Swell2
*T*_0_ (ms)	120.62	80.31	60.22	0	82.89	57.42	28.12	0	120.62	80.62
Vrms (V)	212.67	218.35	220.65	230.54	230.53	230.56	230.42	230.54	251.63	244.18
THD	6.8826	6.8857	6.8821	6.8608	6.8410	6.8852	6.8469	6.8589	6.8784	6.8742
TWD	0.6491	0.6515	0.7078	6.8607	6.8407	6.8848	6.8425	4.6067	0.6564	0.6610
<*ITD*>	0.3279	0.3329	0.3552	6.4397	3.8688	3.3018	2.2395	4.0494	0.3284	0.3409
GDR	0.5257	0.4765	0.4628	6.4397	5.4723	4.2498	2.5544	4.0494	0.5265	0.4783

**Table 5:** Summary of Power Quality Indices for case B.

	Sag	Sag + Harmonics	Harmonics	Harmonics + Transients	Transients
*T*_0_ (ms)	100.62	100.62	0	62.81	62.81
Vrms	200.30	201.51	231.36	232.14	230.79
THD	16.8966	20.2710	10.8804	13.6716	8.2782
TWD	1.2597	11.0999	10.8804	13.6705	8.2766
<*ITD*>	0.7170	10.0536	10.1025	12.2901	4.1376
GDR	1.0778	15.1118	10.1025	16.1499	5.4370

## Data Availability

Not applicable.
